# Silvopasture policy promotion in European Mediterranean areas

**DOI:** 10.1371/journal.pone.0245846

**Published:** 2021-01-22

**Authors:** Francisco Javier Rodríguez-Rigueiro, José Javier Santiago-Freijanes, María Rosa Mosquera-Losada, Marina Castro, Pablo Silva-Losada, Andrea Pisanelli, Anastasia Pantera, Antonio Rigueiro-Rodríguez, Nuria Ferreiro-Domínguez

**Affiliations:** 1 Department of Crop Production and Engineering Projects, High Polytechnic School, University of Santiago de Compostela, Lugo, Spain; 2 Mountain Research Centre (CIMO), Polytechnic Institut of Bragança, Bragança, Portugal; 3 Research Institute on Terrestrial Ecosystems, National Research Council, Porano, Italy; 4 Department of Forestry and Natural Environment Management, Agricultural University of Athens, Karpenissi, Greece; Institute for Sustainable Plant Protection, C.N.R., ITALY

## Abstract

Silvopasture is the deliberate integration of a woody component with grazed pastures as understorey. It is one of the most extended agroforestry practices all over the world. Silvopasture use is key to increase the sustainability of livestock farming systems as silvopasture reduces the use of concentrates since the woody component provides feed for animals. However, it is not an extensively used practice in Europe. This paper aims at evaluating, from Eurostat, LUCAS database and the 118 rural development programs, the current situation of permanent grasslands in the Mediterranean area of Europe as well as the rural development programmes fostering silvopasture to better understand how sustainable land use systems are promoted and provide insights to foster silvopasture across Europe. The results of this study show that most of the policy measures related to silvopasture are adapted to the local necessity. The already existing agroforestry managed land (dehesas/montado) are related to measures supporting regeneration and maintenance while in those areas where agroforestry does not exist the measures are related to forest fire prevention.

## Introduction

Agriculture in Europe is strongly influenced by the Common Agricultural Policy (CAP) which establishes a set of rules for the 28 countries included in the European Union (EU). The CAP consists of two Pillars, the Pillar I (Regulation 1307/2013) where farmers receive direct payments based on the surface they have, and Pillar II (Regulation 1305/2013) which is more linked with the environment because promotes good practices, such as cooperation among producers and environment friendly, climate resilient farming methods. Up to now, the EU provides the general rules for the EU Member States to fund sustainable farming through direct payments across Europe. However, the rules of the Pillar II are usually provided by the Member States to foster sustainable practices according to the different EU biogeographic regions and locally adapt these practices to increase the ecosystem service delivery [1]. The European Commission proposes that the next CAP 2021–2027 be built around nine key objectives which are focused on the three sustainable goals of the United Nations (economic, environment, and social) [2]. The CAP 2021–2027 also recognizes the need for providing sustainable practices that should be adapted at the national level. Therefore, there will not be EU general rules with regard to land use for farmers to receive direct payments. Instead, Member States should fulfil the nine main aims of the CAP and different types of EU strategies (bioeconomy strategy, farm to fork strategy, European green deal…) by demonstrating results linked to key actions such as biodiversity, nutrient efficiency, or climate mitigation.

Grasslands area is one of the most important types of land use in Europe, where, according to the European Statistical Office (EUROSTAT) represents the 50.5 and 18.8% of the whole and agricultural land in Europe (EU-28), respectively [3]. Permanent grassland is usually associated with a permanent soil cover with an important internal dynamic from an ecosystem point of view. Compared with arable lands, grassland areas can sequester more carbon, increase biodiversity, or reduce soil erosion [4]. Permanent grassland definition has been recently modified from a policy perspective. Thus, in the CAP 2007–2013 only herbaceous vegetation was considered as part of the permanent grassland, while in the current CAP (2014–2020) the presence of woody perennials is considered as part of permanent grasslands as a source to feed animals, which turns grasslands with woody perennials eligible to get direct payments in the current 2014–2020 CAP (Regulation 1307/2013) by farmers. Woody perennials are especially relevant in the Mediterranean area of Europe, where herbaceous vegetation is not able to survive during the long summer period, which makes them essential to sustain livestock systems avoiding a huge dependence of external inputs. However, woody perennials can survive these restricted weather periods due to their deep root systems. Moreover, due to the importance of grassland areas as a source of ecosystem services [4], the European Commission included the preservation of this type of land use at the national level as part of the greening (Regulation 1307/2013) while it is part of different programmes of the CAP in the Rural Development Programmes (RDP). If woody perennials are included, permanent grasslands are called silvopasture, a type of agroforestry system able to foster sustainability in rural areas [5,6]. Agroforestry, and therefore silvopasture, presents multiple environmental, economic and social benefits compared with exclusively forest and agricultural systems [6]. For this reason, agroforestry is expanding across Europe despite the lack of technical knowledge transfer and adequate policies promoting agroforestry practices at field level as it is indicated in the conclusions of the EU Agroforestry Innovation Network (AFINET) [7]. In this context, there are 118 RDP in the whole 28 member states, out of which 29 are included in the Mediterranean area of Europe. However, these RDP do not consider in depth the role that agroforestry has to play. Moreover, the Mediterranean area of Europe is one of the most vulnerable regions in the world to the impacts of global warming, which makes necessary to provide policy tools to foster sustainable land use systems in this region of Europe [8]. This paper aims at evaluating the current situation of permanent grasslands in the Mediterranean area of Europe as well as the RDP fostering silvopasture to better understand how sustainable land use systems are promoted and provide insights to foster silvopasture across Europe.

## Material and methods

Results will be presented taking into account the main indicators that affect the productivity of the system and may modify the implementation of policies according to social, geographic, biological and policy aspects. Within the social aspect, the land ownership will be taking into account as an indicator of evaluating long-term practices and as a restriction to receive CAP payment. In the geographic aspect, the altitude will be considered as an indicator of environment constraints. In the biological aspect, the vegetation and the agroforestry practices distribution will be studied. Finally, in the policy aspect, the rural development policies that are developed at the regional level by each member state will be evaluated. These indicators can be related to the promotion of farming systems to fulfil the main pillars of the CAP: social, economic and environmental aspects.

### LUCAS analysis

The way to estimate the silvopasture extent is described in Table 1 [5]. In this study, the “Land use/cover area frame statistical survey”, abbreviated as LUCAS was used to identify silvopasture [5,9]. EUROSTAT has the LUCAS survey micro-data collection of cover and land use which is freely available on the LUCAS website [10]. For this study, we used the LUCAS 2012 data, when Croatia was not part of the EU, so the results are only referred to the EU27.

**Table 1:** Criteria used for identifying the agroforestry (AGF) practices.

Land cover/variable	Code	LUCAS class	Silvopasture AGF	
**Grassland**	E10	Grassland with sparse tree/shrub cover	LC2	
	E20	Grassland without tree/shrub cover	LC2	
	E30	Spontaneously re-vegetated surfaces	LC2	
**Woodland**	C10	Broadleaved woodland	LC1	
	C21	Spruce dominated woodland	LC1	
	C22	Pine dominated woodland	LC1	
	C23	Other coniferous woodland	LC1	
	C31	Spruce dominated mixed woodland	LC1	
	C32	Pine dominated mixed woodland	LC1	
	C33	Other mixed woodland	LC1	
**Permanent industrial crops**	B84k	Mulberries and carob	LC1	
	B84m	Willow	LC1	**LC1** = Primary land cover
				**LC2** = Secondary land cover
**Shrubland**	D10	Shrubland with sparse tree cover	LC1	
	D20	Shrubland without tree cover	LC1	
**Grassland**	E10	Grassland with sparse tree cover	LC1	
**Land management**	1	Signs of grazing	Yes	

LUCAS is a two-stage sample survey. The first phase is a systematic sampling carried out in around 1.1 million points (spaced 2 km). In a second stage, a representative subset of 270,267 points was selected to be physically visited by inspectors.

LUCAS uses a double classification system for land covers that also includes the land use with multiple layers, used only for specific landscapes, such as agroforestry and complex or heterogeneous area. For example, in silvopasture, a woody vegetation layer (LC1) is typically accompanied by the secondary layer (LC2) composed of grass. Another useful variable included in the LUCAS database is land management, which contains information if there are signs of grazing or not, which therefore identify silvopasture. By using LUCAS data we distinguish silvopasture in arable crops (temporary grassland) which are grazed, silvopasture with orchards, and silvopasture within forestland. To estimate the extent of agroforestry of silvopasture in hectares at RDP region level, we divided the number of points coded as silvopasture in each territory by the total number of LUCAS points in this territory and multiplied this by the surface of the territory [5].

### Policy analysis

The policy analysis was conducted considering the measures activated by the Pillar II of the CAP [11] related to the RDP. The methodoly used in this study was previously defined by Mosquera-Losada et al. [5] who carried out a categorization and extent of agroforestry practices linked to agricultural and forest lands at RDP-regional level and evaluate how are they promoted by the previous (2007–2013) and current CAP (2014–2020). Previously, den Herder et al. [12] made the first serious attempt to categorize the extent of agroforestry per country in Europe based on the use of LUCAS and considering the definition of agroforestry in the CAP 2007–2013 framework but not the new definition coming from the deployment of the Measure 8.2 of the Regulation 1305/2017. In this study, a policy analysis evaluating the promotion of agroforestry practices was developed in the deployment of the 29 RDP of the Mediterranean area of Europe in the period 2014–2020 available on the internet and excluding Cyprus and Greece [13]. Moreover, data from Pulla et al. [14] for the forest ownership and from EEA [1] for the altitude were used. We evaluated measures associated with agriculture (temporary grassland which are grazed and orchards) and forest land (usually linked to forest fire risk reduction).

### Upscaling

The obtained geographical indicators from LUCAS (percentage and number of hectares), as well as the policy indicators (activated measures), were upscaled and mapped per region of Europe by using QGIS 2.18. Both forest ownership and altitude have been processed with QGIS 2.18.

## Results

### Social context: The ownership

**Fig 1:**
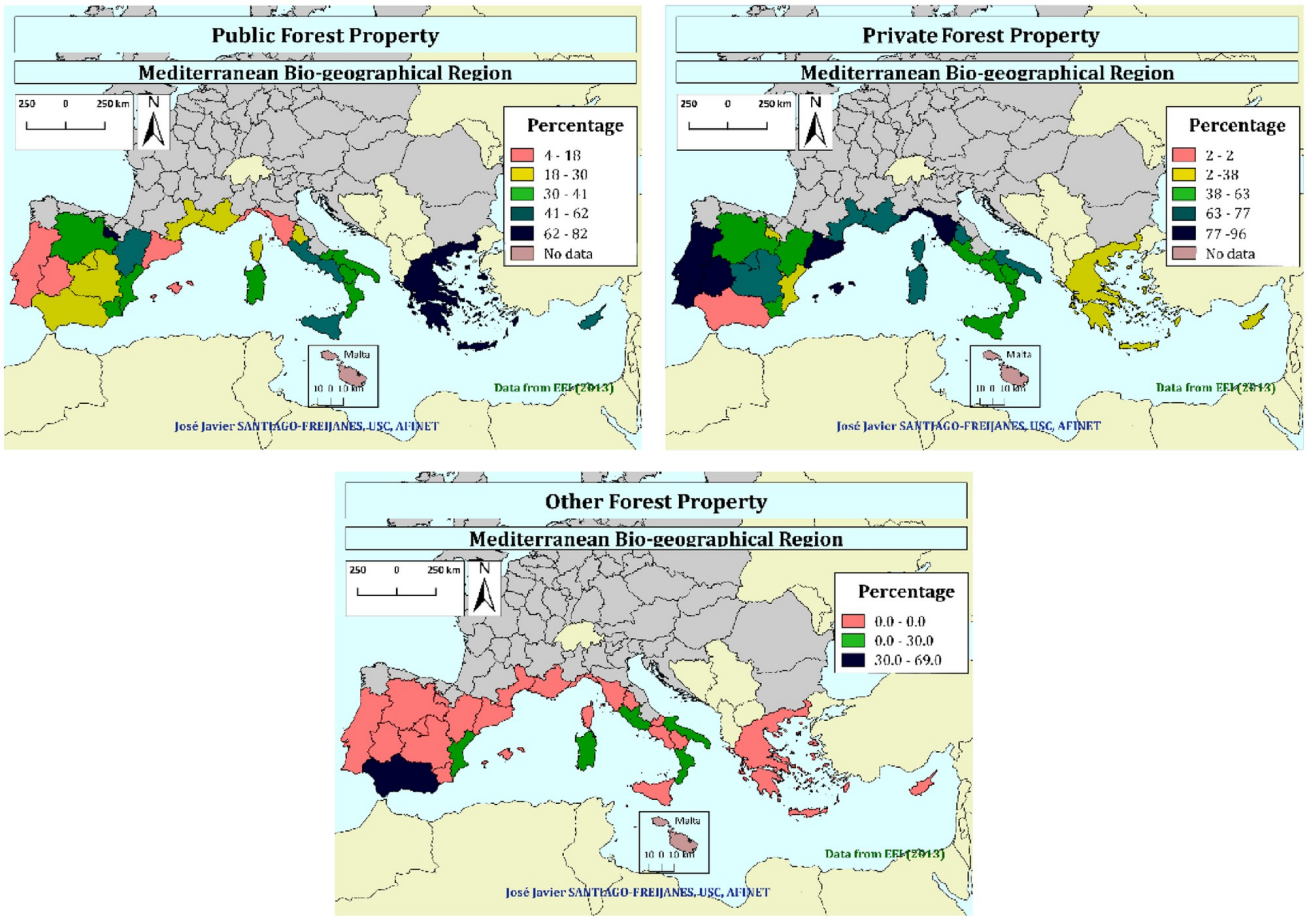
Type of dominant forest property in the European Mediterranean area (public, private, and other type of forest property, following the classification of Pulla et al. [14]).

The type of ownership of forest lands in the Mediterranean Region of Europe is mostly private (Fig 1). Regions like those linked to mainland Portugal, Catalunya, and Extremadura in Spain as well as those like Liguria and Toscana in Italy, have more than 77% of the forest land privately owned.

Other regions placed in Spain have over 30% of public forest (Castilla y León, Aragón, Valencia, Murcia, La Rioja) as happens in Greece, and most of the Mediterranean Regions of Italy excluding Toscana, Umbria, and Sardegna. Only Andalucía has over 30% of the properties associated with “other forest property” being also present to some extent in Italy (Sicilia, Lazio, Puglia, and Calabria) and some other areas of Spain (Valencia). The categorization of other forest ownership depends on each EU Member State, which makes difficult to have a clear comparison of their meaning, but they are usually areas that cannot be categorized either as public or as private.

### Geographic context

**Fig 2:**
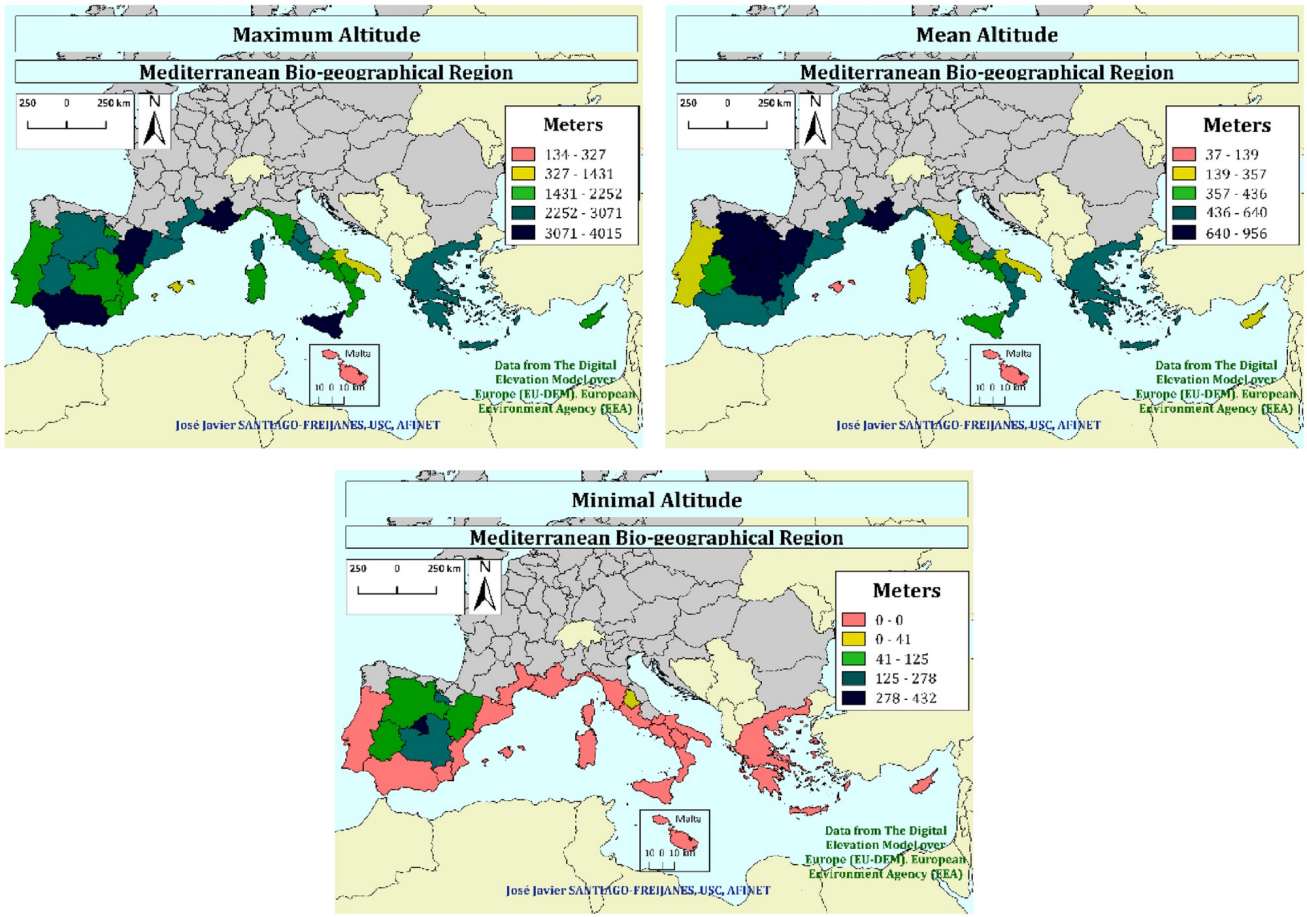
Maximum, mean, and minimum altitude (m asl) of the European Mediterranean regions basin (Data from EEA [1]).

Fig 2 shows the mean, maximum, and minimum altitude of the Mediterranean regions basin. High mountain areas are mainly placed in Spain (Andalucía and Aragón), France (Provence-Alpes-Côte d’Azur), and Italian (Sicilia). The maximum mean altitude is observed in the Central Spanish plateau, and France (Provence-Alpes-Côte d’Azur). The lowest mean altitude is found in most of the Mediterranean countries except for the Spanish Plateau.

### Mediterranean agriculture and woodland

**Fig 3:**
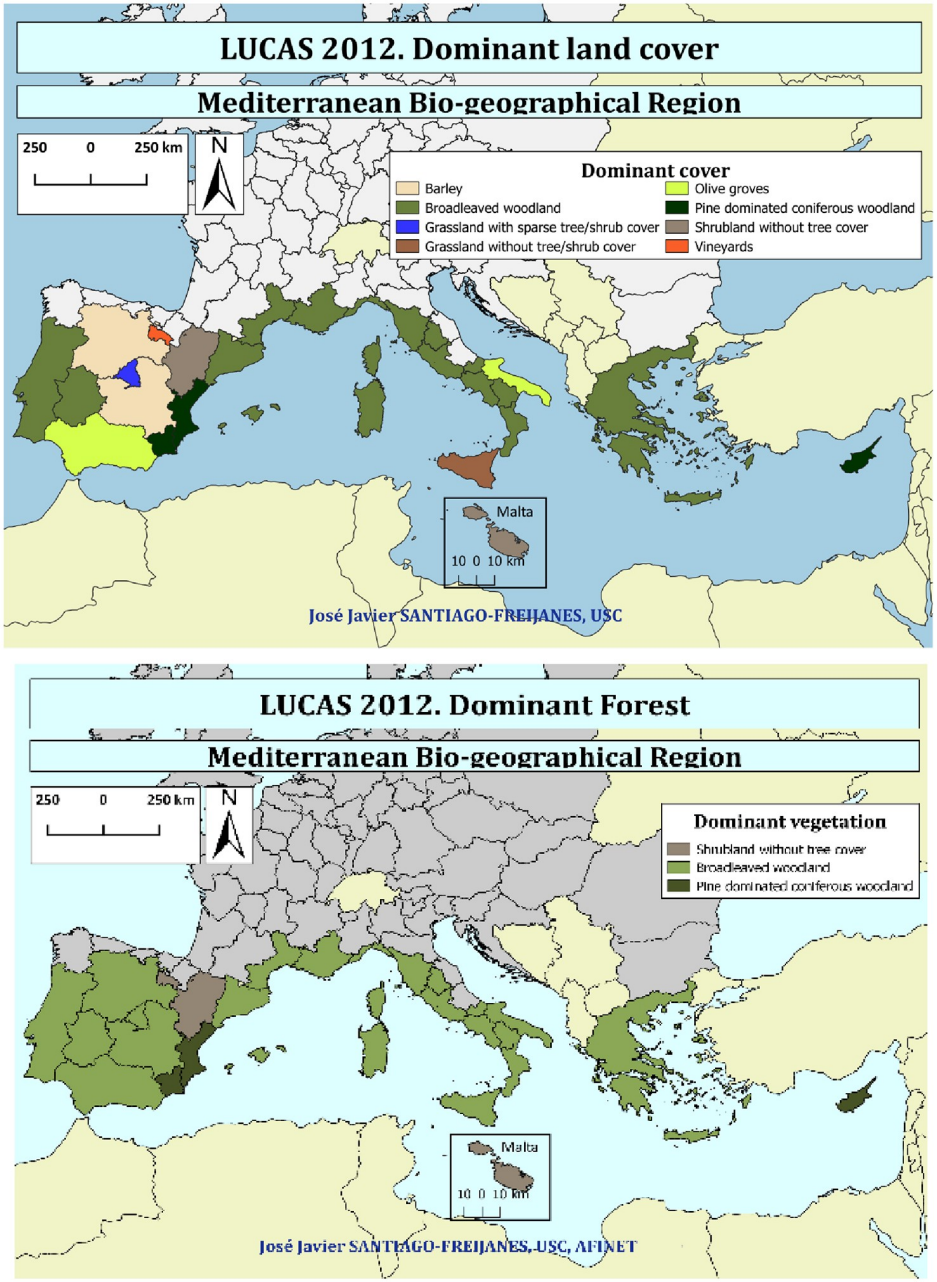
Share of dominant land cover, in the Mediterranean regions of Europe.

Fig 3 shows the dominant type of land cover in the regions of the Mediterranean area of Europe. Grassland without tree/shrub cover is the dominant land cover in Sicilia, an area with a high altitude. The rest of the regions are mainly dominated by woody vegetation such as grassland with sparse tree/shrub cover (Madrid), olive groves (Andalucía), shrublands without tree cover (Aragón and Malta), and broadleaved woodlands. Pine dominated conifer land use is mainly placed in Valencia, Murcia, and Cyprus.

If the forestland use is considered (Fig 3), most of the European Mediterranean area is dominated by broadleaved woodlands. Murcia and Valencia regions in Spain, together with Cyprus, are associated with Pinus dominated coniferous woodland. Regions with a high proportion of public property such as La Rioja and Aragón in Spain and Malta are mainly associated with shrubland without tree cover. However, the size of the shrubs and the trees may vary a lot, and sometimes it is difficult to establish a clear criterion to easily distinguish these two types of vegetation due to the different definitions among countries.

### Mediterranean agroforestry

**Fig 4:**
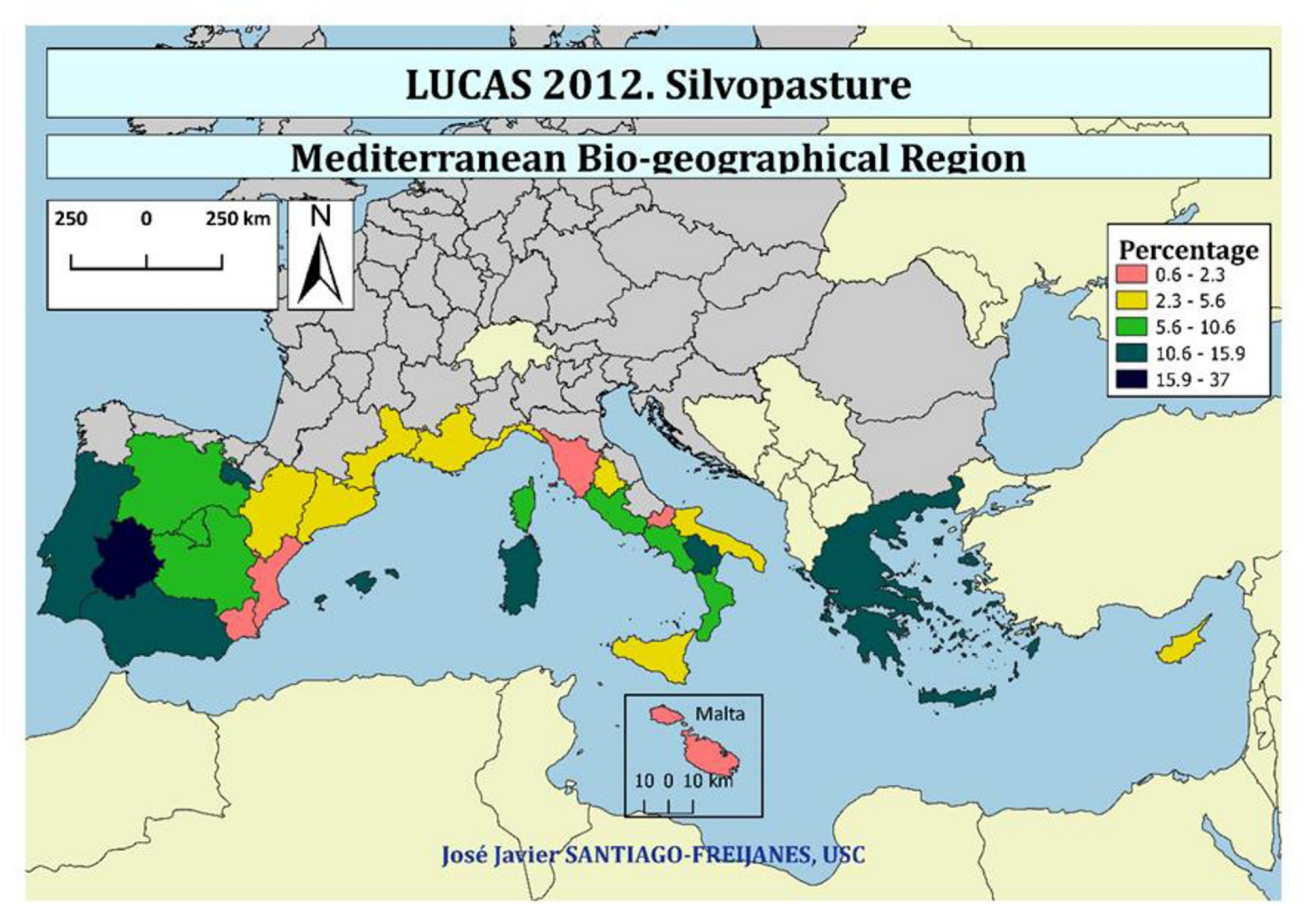
Percentage of land occupied by silvopasture.

Silvopasture is the most important agroforestry practice in Europe, reaching up to 37% of the land in some regions of Europe (Fig 4). Silvopasture is mainly located in Extremadura, La Rioja, Baleares, and Andalucia in Spain, Sardegna, and Basilicata in Italy besides Portugal and Greece. On the contrary, the Spanish regions of Murcia and Valencia in Spain, Toscana, and Molise in Italy, as well as Malta, have the lowest share of silvopasture in the Mediterranean area of Europe.

**Fig 5:**
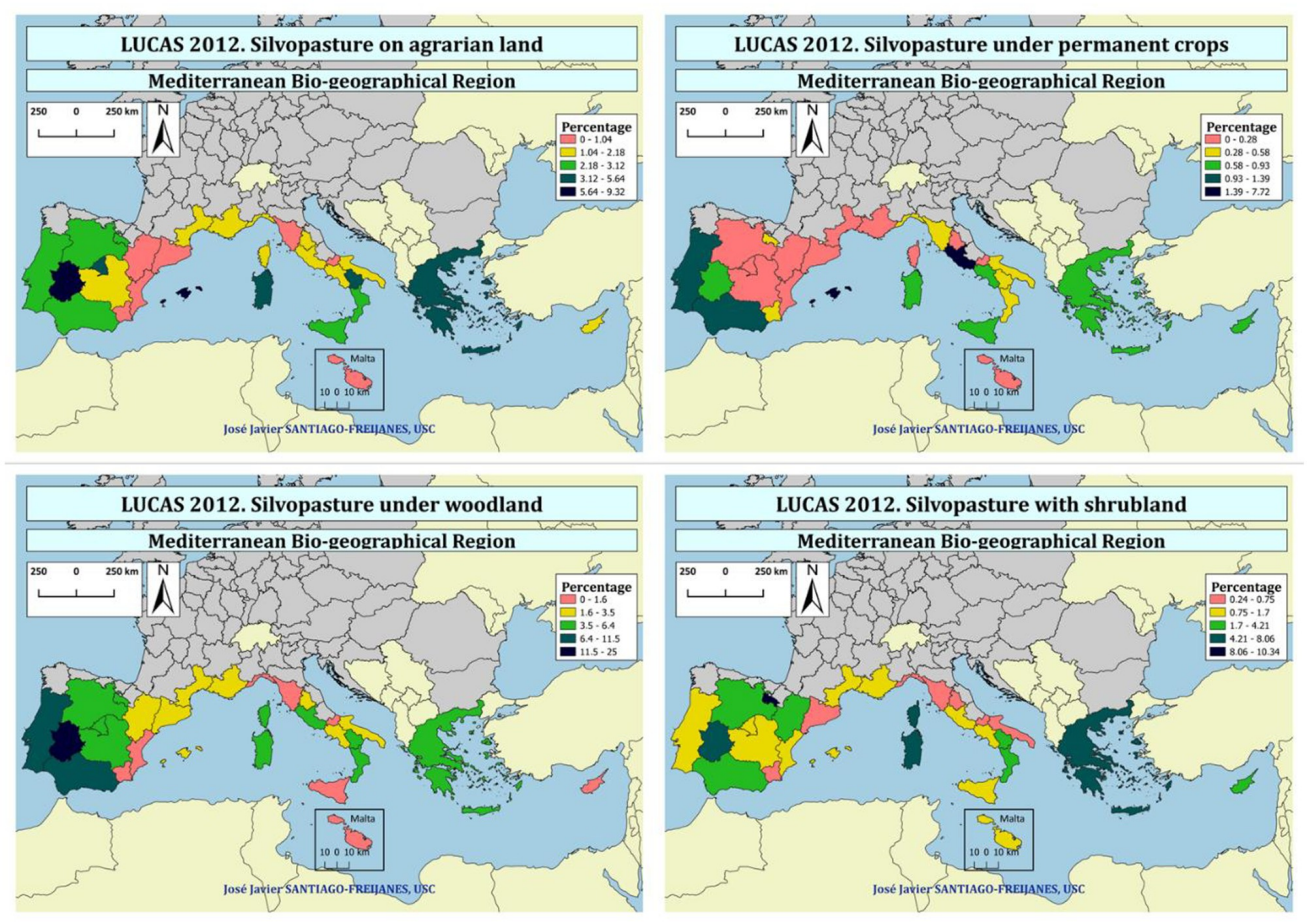
Percentage of silvopasture (pasture) in agrarian lands (the sum of (i) pastures with non permanent crop trees and grazed pasture as understory and (ii) permanent crop (fruit + nut) trees and grazed pasture as understory), and lands linked to woodland and shrublands.

Silvopasture can be part of agricultural lands as well as forestlands mainly dominated by woodlands or shrublands as shown in Fig 5. Pasture linked to permanent crops are dominant in Baleares and Andalucia with a high share of olive trees, but also in Portugal and Lazio in Italy. However, when silvopasture linked to no fruit trees is joined to those linked to fruit trees, is Extremadura, Madrid, Baleares in Spain and Basilicata and Sardegna in Italy, as well as Greece the ones with the higher share or silvopasture in agricultural lands.

Pasture under shrubland is mainly associated with La Rioja, the Spanish Dehesa (Extremadura), Sardegna (Italy), Corsica (France) and Greece followed by some regions of Italy (Lazio, Basilicata, and Calabria), Spain (Andalucia, Castilla y León, and Aragón). When silvopasture linked to woodlands is evaluated, Extremadura but also Andalucia and Portugal, the places with the largest share of the Iberian present the highest share, followed by Castilla León, Madrid, La Rioja and Castilla la Mancha in Spain, Corsica in France, Sardegna, Basilicata, Calabria and Lazio in Italy and also Greece.

### Policy

**Fig 6:**
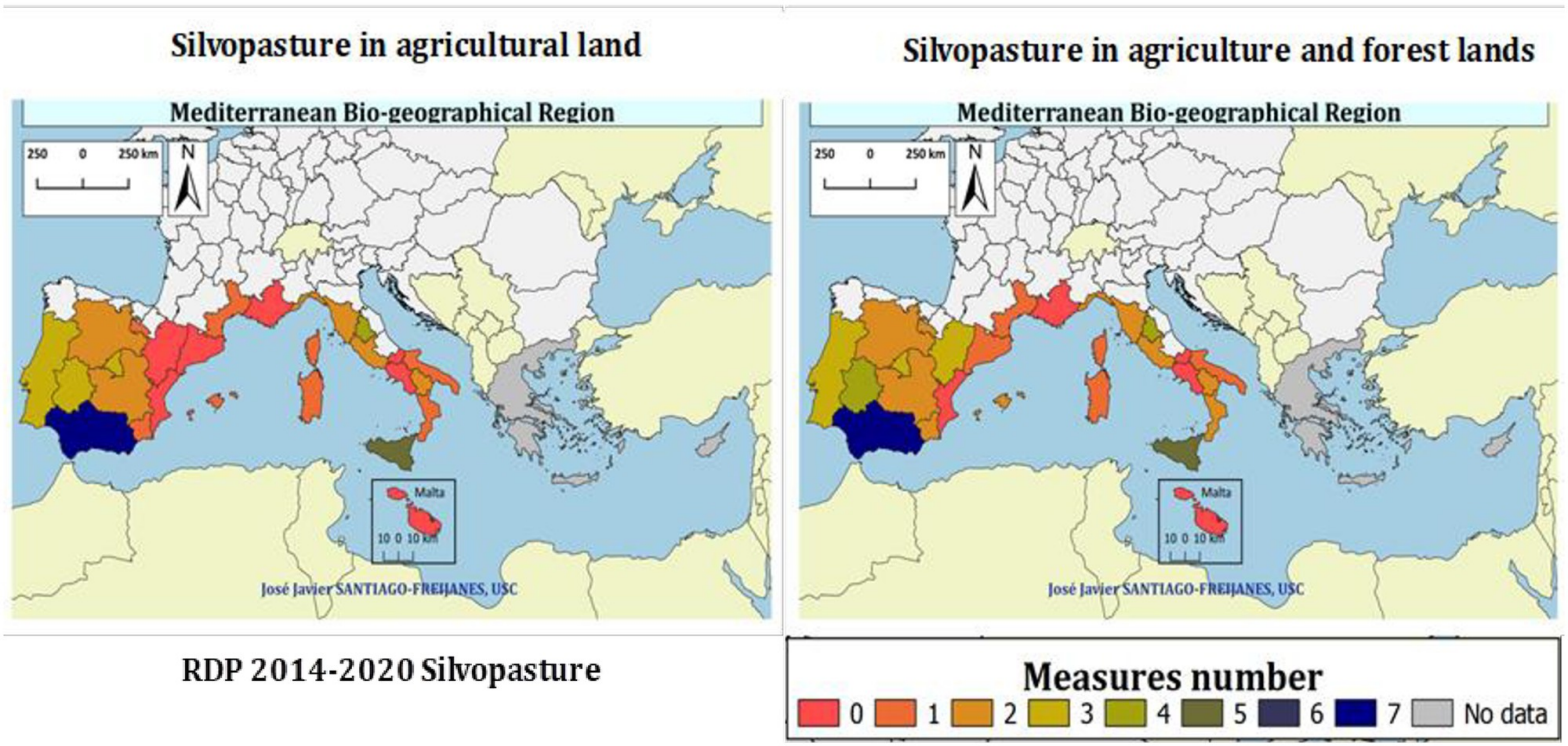
Number of Rural Development Programmes (RDP) 2014–2020 measures promoting silvopasture with annual cropland forest farming.

Fig 6 shows the number of RDP measures that promote the different types of silvopasture within the 2014–2020 RDP in both agricultural and forest lands. Regarding the number of measures promoting silvopasture with temporary grassland (arable lands), there are nine regions not promoting silvopasture. Most of the Mediterranean regions promote silvopasture with one measure, while Andalucia uses seven measures and Sicilia uses five. Four measures are implemented in Umbria and three in Madrid and Extremadura, in Spain, and Portugal.

Table 2 shows the type of silvopasture measures per region to improve silvopasture management, establishment, and improvement. Agroforestry education is promoted through measures 1.2 with the establishment of demo sites and with measures 2.1 and 2.3 by fostering farmers and advisors knowledge about agroforestry in the Andalucia RDP. Silvopasture is also promoted with Measures 4.1, 4.3, and 4.4 through the development of investments in physical assets such as regeneration in five Spanish RDP. Moreover, silvopasture is also promoted in forest areas by the agroforestry measure 8.2 itself, aiming at establishing and improve silvopasture management in Portugal, two Spanish RDP (Andalucia and Extremadura), and three Italian RDP (Puglia and Basilicata). Silvopasture establishment and improvement are also carried out by measure 8.3 (Castilla and León) to reduce fire risk, 8.5 (Basilicata, Toscana, and Lazio) to improve the forest resilience, and 8.6 (Toscana and Umbria) to mobilize silvopasture products. Silvopasture is also enhanced by measures 12.1 to improve Nature 2000 areas and 15.2 to preserve forest areas. Agri-environment measure 10.1 is deployed to improve silvopasture management and establishment in the rural development programs of Spain (La Rioja, Madrid, and Andalucia), France (Corse), Italy (Sardegna and Lazio), and Portugal, but also in Castilla la Mancha through the promotion of grazing with landscape features. Silvopasture is also supported with permanent crops, namely, orchards through measure 10.1 in Spain (Castilla y León), France (Languedoc), Italy (Liguria, Calabria, and Sicilia), and Portugal, but also through measures 8.6 and 11.1 in Italy (Toscana and Sicilia).

**Table 2:** Measures favouring silvopasture combined with woody perennials and orchards. RDP: Rural Development Program; Reg: Regeneration.

Country	RDP	Silvopasture	Orchard silvopasture	Country	RDP	Silvopasture management, establishment, and improvement	Orchard silvopasture
**Portugal**	**Portugal**	8.2, 10.1	10.1	**Italy**	**ITC3 Liguria**	12.2	10.1
**Greece**	**Greece**				**IFF4 Puglia**	8.2	
**Spain**	**ES23 La Rioja**	10.1			**ITF5 Basilicata**	8.2, 8.5	
	**ES30 Madrid**	4.4, 8.3, 10.1			**ITF6 Calabria**		10.1
	**ES41 Castilla y León**	8.3	10.1		**ITG1 Sicilia**	8.3, 8.4, 8.5	10.1, 11.1
	**ES42 Castilla la Mancha**	4.3, 10.1 (LF)			**ITG2 Sardegna**	10.1	
	**ES43 Extremadura**	4.3, 4.4 (reg), 8.2 (reg)			**ITI1 Toscana**	8.5, 8.6	8.6
	**ES61 Andalucía**	1.2 (demo), 2.1(farmers assessment), 2.3 (advisor assessment), 4.1, 4.4 (reg), 8.2, 10.1			**ITI2 Umbria**	8.2, 8.6, 12.1, 15.2	
	**ES62 Murcia**	4.1			**ITI4 Lazio**	8.5, 10.1	
	**ES63 Baleares**	1.2 Demo					
**France**	**FR83 Corse**	10.1					
	**FR81 Languedoc**		10.1				

One of the most important aspects to foster agroforestry is the development of management plans (Table 3) in general, as promoted by measure 11.2 in La Rioja or measures 8.1 and 8.4 in Aragón. Also, management plans focused on (i) risk prevention as carried out in Spain by measures 8.1 and 1.2 in Extremadura and Andalucía, respectively, and by measure 8.3 in Spain (Aragón, Madrid, Cataluña, and Murcia) and Italy (Sicilia) (ii) restoration fostered by measures 8.3 in Spain (Baleares), Italy (Calabria) and Portugal, and 8.4 in Spain (Aragón) and Italy (Sicilia) and (iii) disaster prevention developed by measure 8.3 in Aragón.

**Table 3:** Woodland production through the development of forestry technologies, processing, mobilising, and marketing of forest products as well the value chain per rural development program (RDP).

		Agroforestry management plans				No Timber Woodland Products (NTWP)		
Country	RDP	General	Forest fire prevention	Forest restoration	Country	RDP	NTWP value chain	NTWP production
**Spain**	**ES23 La Rioja**	11.2			**Spain**	**ES23 La Rioja**		8.6
	**ES24 Aragón**	8.1, 8.4	8.3	8.4		**ES42 Castilla la Mancha**	9.1	
	**ES30 Madrid**		8.3			**ES52 Valencia**	8.6	
	**ES43 Extremadura**		8.1			**ES43 Extremadura**		8.6
	**ES61 Andalucía**		1.2			**ES61 Andalucía**	1.2	1.2, 8.6
	**ES53 Baleares**			8.3				
	**ES51 Cataluña**		8.3					
	**ES62 Murcia**		8.3		**Italy**	**ITF3 Campania**	4.1	
**Italy**	**ITF6 Calabria**			8.3		**ITG2 Sardegna**	8.6	
	**ITG1 Sicilia**		8.3	8.4		**ITI2 Umbria**	8.6	
**Portugal**	**Portugal**			8.3	**Portugal**	**Portugal**	4.2	8.6

Both no timber woodland production through the development of forestry technologies, processing, mobilizing, and marketing of forest products, as well as the development of the value chain, are key to increase diversification and income for farmers from forestlands (Table 3). The forestry technologies related to processing, mobilizing, and marketing of forest products are mostly fostered through measure 8.6 in Spain (La Rioja, Extremadura, and Andalucia which also uses M1.2) and Portugal. Value chain improvement is key to foster agroforestry associated to forest lands as recognized in Spain (La Rioja and Valencia through measure M8.6, Castilla la Mancha (M9.1) and Andalucia (M1.2)), Italy (Campania (M4.1) and Sardegna and Umbria through measure 8.6) and Portugal by financing investments (M4.2).

## Discussion

The Mediterranean area of Europe is characterized by mild temperatures on winter but hot temperatures and lack of precipitation in summer, which may explain perennial crops as permanent grasslands or permanent crops (mostly olive groves and in some Spanish areas nut trees and/or vineyards) are the dominant vegetation in this area. Depending on the woody perennials, silvopasture could be related to forestlands (oaklands, shrublands, and pine stands) or agricultural lands (low tree density or permanent crops (e.g. fruit trees)). The dominant woody perennial vegetation delineates the first framework for the development of the silvopasture agroforestry systems. Silvopasture implementation is a type of seminatural system where management transforms the landscape. Anthropogenic pressure, linked to intense pastoral and arable activities in the Mediterranean forest, caused a reduction in pine-oak forests in the Mediterranean area for centuries, being this reduction more intense in the second half of the XXth century [15]. Afterwards, land abandonment and the EU and National policies linked to both reforestation and afforestation have increased the proportion of forestlands as the dominant vegetation in most Mediterranean regions [16]. Moreover, farm abandonment in the Mediterranean areas associated to land degradation, water scarcity linked to climate change, and depopulation associated to migration from rural to urban areas have caused a natural expansion of unmanaged forestlands in most of the European regions of the Mediterranean area [17,18] leading to a rise on forest fires [19,20]. In the west part of the Mediterranean area of Europe, there are also well-managed oaklands dominating some landscape regions as part of the most important agroforestry system associated to livestock production: the dehesa. The dehesa is recognized as an example of land use sustainability and a hotspot of biodiversity and resilience while having a low forest fire risk. The positive income and the high number of ecosystem services delivered by the dehesa/montado made both Extremadura and Portugal have the lowest share of public ownership of oaklands in Europe [21]. This low public ownership of oaklands also occurs in the North of Spain (Cataluña) as well as French Mediterranean regions due to the high population density they have and the negative impact that anthropogenic pressure causes on land use through the implementation of agriculture in the Mediterranean ecosystem. In this context, previous studies have highlighted that the land ownership regime has a clear influence on the type of land mangment [22]. Moreover, the dehesa area has the largest share of silvopasture of the Mediterranean region linked to agricultural land including permanent crops and also to forest lands where grazing is part of the shrublands and woodlands. The large share of agroforestry in the dehesa systems makes the number of policy measures associated with this land use very high compared with most of the regions in the Mediterranean part of Europe. Measures linked to dehesas are associated to dehesa regeneration in the forest and agricultural lands due to the age of the trees which are several centuries old but also to the lack of regeneration associated to inadequate grazing management and climate change which is currently causing huge mortality in oaks [23–25]. The protection of dehesas in Spain and Portugal makes also important the policy support associated with the agri-environmental measures due to the ecosystem services the dehesa deliver [26]. One of the reason of the dehesa success is the already developed excellent supply chain strategy it has linked to the “Iberian pig”. This commercialization success makes the value chain measures not relevant in this part of the Mediterranean area of Europe, being mostly linked to other non-timber woodland production in both Portugal and Extremadura to increase the multiple-use and products obtained from the system (e.g. mushrooms, honey…).

Opposite to the well designed and managed dehesa systems, adapted to the Mediterranean weather conditions, there are other areas with high anthropogenic pressure in the past reflecting a high degree of degradation [17,18]. These degraded areas were mostly reforested by using pioneer tree species such as pine to protect the soil against erosion, as happened in Murcia, Valencia, and Cyprus areas, currently dominated by pine species with a low rate of silvopasture implementation. The presence of conifer plantations in these areas is usually linked to marginal, degraded, and high altitude areas and very poor soils. Conifers provide a higher level of carbon sequestration than agricultural lands [27]. but less benefit for silvopasture practices than oaklands due to the less shade (animal welfare) and feed resources (acorns) that pines provide compared to oaks [28]. Pine plantations are more sensitive to drought stress and provide fewer ecosystem services than oaklands [29,30]. Oaklands are extensively dominating the Mediterranean area of Europe where the number of agroforestry measures is small. As mentioned, land abandonment caused an oakland expansion in most of the European regions prone to be fired in Mediterranean weather conditions [17,18]. This justifies the large number of regions implementing silvopasture through the forest policy measure 8.3 associated with forest fires fighting. Measure 8.3 aims at reducing the understory as a forest fuel. Some regions also implement measures associated with forest restoration after fires happened. Silvopasture and forest farming promotion in Mediterranean areas should be based on good agroforestry management plans founded on the local conditions, but also on the improvement of the production and resilience and the promotion of value chains as challenges highlighted by the 1500 stakeholders participating the EU thematic network Agroforestry Innovation Network (AFINET) [7]. Most of the Italian and French regions present a shortage of agroforestry practices linked to silvopasture, except for Calabria and Basilicata, where agroforestry is promoted by the introduction of agroforestry systems as part of the measures 8.2 and 9.5. Despite the low share of silvopasture in woodlands and shrublands of the French and Italian regions, compared with other regions of the Mediterranean area of Europe, most of the regions have measures linked to the establishment and maintenance of different forms of silvopasture in forest areas but also associated to the development of value chains as in Umbria and Sardegna regions. Greece is one of the regions with a large share of grazed shrublands, probably because shrubs fit as a source of feed for the largest density of goats of the European countries that Greece has [31].

Aragón, Malta, and La Rioja are the three regions with a land cover dominated by unmanaged shrublands, as the share of silvopasture is rather low but higher than in other areas of Europe not dominated by shrublands. Unmanaged shrublands are transformed into forests [17,18] while fired forests are usually transformed in open shrublands [32]. This promotes that only La Rioja has allocated measures to improve the production of no timber woodland products and the Agri-environment measure to protect these systems. Silvopasture linked to permanent crops is indeed relevant in areas where olive trees represent a large share of the region such as Andalucia and Puglia, but also Portugal, Basilicata, and Lazio. The importance of agroforestry practices within the olive orchards is linked to the intensive farming system they have suffered in the last decades with important soil erosion and degradation that can be recovered by sowing pasture under the olive trees. Furthermore, livestock grazing increases the preservation of the olive soil stands as a sustainable way to reduce competition with trees while favouring nutrient recycling through faeces and urine deposition. Andalucia has devoted measures to increase farmers knowledge on silvopasture systems, with both permanent crops and woodlands (to reduce forest fire risk), through the implementation of measures related to demo sites and farmers and advisor assessment, as highlights the EIP-Agri [33] innovation development schemes. The establishment of agroforestry demo-sites linked to the RDP was also implemented by the Baleares Islands region, where nut trees are dominant.

Mediterranean islands acknowledge a different share of land use cover, from those with a high anthropogenic impact (Sicily) to those with lower impact (Sardegna or Corsica). Sicily is closer to the continent than the other Mediterranean islands and presents a long tradition of olive trees agroforestry, declining nowadays due to the land abandonment [34]. However, it is currently still possible to find agroforestry areas as traditional cultural landscapes [35]. On the contrary Sardinia, with less anthropogenic impact linked to a lower population density and connectivity with the continent, is managed to maintain traditional silvopasture practices linked to the biodiversity hotspot of this island. Land abandonment in the last decades has conducted to a clear reduction of silvopasture practices and an increase in forest lands [36]. Similarly, still, some agroforestry and an increase of forest lands can be found in Corsica [37], Cyprus [38], and Baleares especially linked to nut production. Agroforestry policy measures in the Mediterranean areas are associated with demo sites and forest prevention techniques in Baleares, and forest and agri-environment measures in Sicilia and Sardegna. Value chain promotion is activated as an RDP measure in Sardegna.

The presence of woody perennials in most of the Mediterranean area of Europe can be related to the fact that deep-rooted species are needed to overcome the long and dry summer that Mediterranean plants have to face while providing feed to animals in silvopasture systems [39]. Both the deep-rooted perennials and the sward annual species of the permanent grasslands can be considered as climate adaptation mechanisms traditionally existing in this part of Europe where precipitation intra-annual variability is so frequent [40]. Shrublands and small trees are the main source of feed for the small domestic mammals of Southern Europe (goats and sheep) during most of the year and especially in the summertime. The introduction of deep-rooted perennials in agricultural systems is one of the recommendations of the European Commission EU as part of the indicative measures that may be included in the information on Land use, land-use change, and forestry (LULUCF) actions submitted under Article 10(2)(d) (Decision 529/2013/EU) that can be related to agroforestry. The highest proportion of permanent grasslands in the South of Europe is associated with a better adaptation of grasses than arable crops to the Mediterranean lack of water [40].

## Conclusion

Silvopasture is an important practice across the Mediterranean region, mostly associated with oaklands, but also present in permanent crops (olive) in some areas. The extent of silvopasture is high in the west part of the Iberian Peninsula where the share of public land is low as financial benefits are obtained from the land. However, most of the regions have a low extent of silvopasture and can be linked to a high (intensive agriculture) and low (abandonment) anthropogenic pressure. Most of the policy measures related to silvopasture are adapted to the local necessity. The already existing agroforestry managed land (dehesas/montado) are related to measures supporting regeneration and maintenance while in those areas where agroforestry does not exist the measures are related to forest fire prevention.
